# Acceptability and feasibility of delegating HIV counseling and testing for TB patients to community health workers in the Philippines: a mixed methods study

**DOI:** 10.1186/s12889-019-6497-7

**Published:** 2019-02-13

**Authors:** Tyrone Reden L. Sy, Retna Siwi Padmawati, Emmanuel S. Baja, Riris Andono Ahmad

**Affiliations:** 1 Department of Biostatistics, Epidemiology, and Population Health, Faculty of Medicine, Public Health and Nursing, Universitas Gadjah Mada, Yogyakarta City, 55281 Indonesia; 2 Department of Health Behavior, Environment and Social Medicine, Faculty of Medicine, Public Health and Nursing, Universitas Gadjah Mada, Yogyakarta City, 55281 Indonesia; 3 Department of Clinical Epidemiology, College of Medicine, University of the Philippines Manila, Manila City, Philippines; 4 Institute of Clinical Epidemiology, National Institutes of Health, University of the Philippines Manila, Manila City, Philippines; 5 Center for Tropical Medicine, Faculty of Medicine, Public Health and Nursing, Universitas Gadjah Mada, Yogyakarta City, 55281 Indonesia

**Keywords:** Task shifting, Patient-centered care, TB/HIV, Implementation research, Barangay health workers, Appropriateness, PITC, Opt-out HCT

## Abstract

**Background:**

The Philippines has a high burden of TB and HIV, yet the WHO estimates that only 13% of Filipino TB patients know their HIV status. This is partly attributable to the lack of trained HIV counselors and medical technologists (or laboratory technicians) at the primary healthcare level. In Africa where resources and manpower are also scarce, TB/HIV care is already delegated to community health workers. Evidence is scant however about the acceptability and feasibility of engaging community health workers to provide HIV counseling and testing (HCT) among TB patients in the Philippines. The objective of this paper is to describe and assess the acceptability and feasibility of delegating HCT among TB patients to *barangay* (community) health workers (BHWs) in the Philippines.

**Methods:**

Mixed methods study that utilized surveys with patients (*n* = 89), BHWs (*n* = 81), and ten focus group discussions with key stakeholders (*n* = 49) in San Jose del Monte, a city with high HIV prevalence. A facility assessment (*n* = 17) was done using a modified version of WHO-Service Availability and Readiness Assessment questionnaire to assess feasibility (scale of 1 to 4) while acceptability from the perspective of patients and BHWs was determined using surveys.

**Results:**

Only 47% of TB patients agreed to receive HIV counseling from BHWs, while 30% agreed to receive HIV testing. Doctors were preferred by patients as HIV counselors, while medical technologists were preferred as HIV test providers. Two out of three BHWs also disagreed to provide HCT but the same number felt that they could provide HCT if additional trainings were given to them. In the group discussions, BHWs preferred to only provide HIV counseling. Stakeholders said that only select BHWs who meet certain criteria should provide HIV counseling. Program managers and stakeholders rated delegation of HCT to BHWs as moderately feasible.

**Conclusions:**

Delegation of both HIV counseling and testing among TB patients to BHWs is feasible, but only delegation of HIV counseling is acceptable. Further studies are needed to guide revision of Philippine HCT policies to accommodate lay HIV counselors, and strengthen the mechanisms for delivering integrated TB and HIV services especially at the primary healthcare level.

**Electronic supplementary material:**

The online version of this article (10.1186/s12889-019-6497-7) contains supplementary material, which is available to authorized users.

## Background

According to the Joint United Nations Programme on HIV/AIDS (UNAIDS), the Philippines has the highest HIV infection growth rate in Asia and the Pacific [1]. In 2016, there were 10,500 new infections recorded, up by 140% from the 4300 cases in 2010. Indeed, the HIV/AIDS and ART Registry of the Department of Health (DOH) Philippines reported that there are 29 newly diagnosed HIV cases per day, up from 26 per day in 2016, and 17 per day in 2014, mostly among males having sex with males (MSMs) aged 25 to 34 years old [2]. The UNAIDS further estimates that only 67% of people living with HIV know their status, and 32% who know their status are on treatment [1].

The Philippines is also one of the 30 countries with a high burden of tuberculosis. Preliminary results of the recently concluded National TB Prevalence Survey in the Philippines found that there are 554 cases of TB per 100,000 population [3], above the World Health Organization’s 2015 estimates of around 322 TB cases per 100,000, and 4.3 HIV + TB cases per 100,000 [4]. Despite the high burden of TB and HIV in the country, WHO estimates that only 13% of TB patients know their HIV status. In part, the DOH-Philippines attributes this to the insufficient number, inequitable distribution, and fast turnover of HIV testing-proficient medical technologists (or laboratory technicians) and DOH-trained HIV counselors at the primary healthcare centers [5].

In sub-saharan Africa where TB and HIV are prevalent and where there is also limited manpower and financial resources, community health workers have been tapped as manpower complements to provide TB/HIV care [6, 7]. Thus, one possible solution to the scarcity of trained providers is to delegate HIV counseling and testing (HCT) to *barangay* (community) health workers or BHWs. While other studies have found that BHWs can be successfully enlisted in sputum collection and malaria immunochromatographic tests [8, 9], there is scant evidence about the feasibility and acceptability of delegating HCT among TB patients to BHWs especially in a devolved health system where local government executives have the power to decide health priorities [10]. The present study aims to describe the acceptability and feasibility of delegating HCT among TB patients to BHWs in the Philippines.

## Methods

### Aim, design and study settings

This is a mixed-methods study which included face-to-face surveys with TB patients and BHWs, focus group discussions (FGDs) with program managers, non-government organizations (NGOs), patient organizations, and primary healthcare center personnel involved in TB/HIV service delivery in the Philippines. An assessment of primary healthcare facilities was also done.

The survey was conducted in primary health centers (*n* = 17) in San Jose del Monte City (SJDM), Bulacan province, Luzon island, Philippines. SJDM is a Category A high HIV prevalence (3.57%) city with a TB incidence rate of 269 per 100,000 and a TB + HIV co-infection rate of 1.1 per 100,000. A significant portion of the population in this City are urban poor informal settlers relocated from Manila and Quezon City, both of which are high-HIV prevalence areas.

### Sampling design

The sample size calculation (*n* = 88) was estimated to be able to detect that 35% of TB patients would agree to household HIV testing based on a study in South Africa [11], with 95% confidence level and 10% precision. TB patients included in the study were those clinically-confirmed to have pulmonary TB and are at least 18 years old. Patients were not approached directly; they were invited to participate in the study through health providers who utilized a prepared invitation script. Patients who have expressed willingness to participate in a brief interview post-survey (*n* = 17, 19%) were interviewed by the first author to clarify their answers especially to the items on appropriateness of delegating HCT to BHWs.

The number of BHWs (*n* = 81) who participated in the survey were proportionally selected based on the size of the health centers. BHWs who have been working as a BHW in the past six (6) months, and who volunteered to participate during the study team’s facility assessment visits were included in the final sample.

Ten FGDs were conducted including two for primary healthcare personnel (*n* = 26), two for BHW leaders (*n* = 16), three for program managers (*n* = 16), and three for NGOs (*n* = 17). The FGDs for primary healthcare personnel, program managers, and NGO representatives were done to assess their perceptions of acceptability and feasibility of delegating HCT to BHWs while FGDs with BHWs themselves were used to triangulate the findings of the survey. The FGDs also elicited the participants’ views regarding requirements for delegation of HCT to BHWs to be feasible. The participants in most FGD were homogenous in terms of profession / characteristics, however one FGD was composed of six physicians and one medical technologist. Primary data collection was done from March to April 2017. The first author and a trained research assistant conducted the FGDs and the surveys.

### Data collection

#### Feasibility assessment

During the FGDs, stakeholders and program managers were asked what the various requirements necessary to make the delegation of HCT to BHWs feasible at the primary healthcare level were. The inputs from the FGDs were used to modify the TB and HIV modules of the WHO-Service Availability and Readiness Assessment [12], and this was used to conduct facility assessments in SJDM (Quantitative Instrument 2, page 13, Additional file 1). Results of the facility assessments were then compared with the feasibility requirements identified, and program managers at the national, provincial, regional and city level were asked to rate each requirement from 1 (low feasibility), 2 (moderately low feasibility), 3 (moderately high feasibility), and 4 (high feasibility). (Quantitative Instrument 4, page 26, Additional file 1). The investigators developed this feasibility scoring scale in consultation with the FGD participants and the program managers.

#### Acceptability assessment

Acceptability from the patients’ perspective was calculated as proportion of respondents who answered ‘agree’ ‘strongly agree’ and ‘very strongly agree’ to questions 1 to 10 of the patient survey (page 24, Additional file 1). Acceptability from BHWs’ perspective was calculated as proportion of respondents who answered ‘agree’ ‘strongly agree’ and ‘very strongly agree’ to question 1 of BHW survey (page 9, Additional file 1). The survey questionnaires were pretested in Manila City, also a Category A city similar to San Jose del Monte. During the pretests, patients preferred to have separate answers for items that assessed appropriateness of receiving counseling and testing from various healthcare providers. Conversely, BHWs preferred that they be asked for the acceptability of becoming both HIV counseling and testing providers per local regulations.

Both quantitative and qualitative data collection tools can be found in Additional file 1. Questions in both tools were sourced from previous researches [13–15] and initial informal discussions with program managers and potential respondents. Two international experts on TB and HIV research were then consulted to further ensure the face and content validity of the questions to be included in the instruments. Likert scales in the patients’ and BHWs’ questionnaire had a Cronbach’s Alpha of .79 and .93, respectively.

### Data analysis

FGDs were recorded using a voice recorder and transcribed verbatim in Filipino using InqScribe Software. A two-phase validation of the transcription was done: first, a different transcriber checked the transcriptions with the audio files, and the principal investigator validated the transcriptions again. The transcribers, proficient in both English and Filipino, translated the transcripts to English. The first author conducted thematic coding of the transcripts (e.g. coding significant sentences vis-à-vis the research objectives, then clustering codes into themes). The codes and themes were then discussed and validated with the co-authors. OpenCode 4.03 was used to assist in qualitative data analysis. Stata for Macintosh version 11.0 was used to run descriptive statistics.

### Ethical consideration

The study was given ethical approval by the University of the Philippines Manila Research Ethics Board (Decision Letter 2017–045-01). All respondents were given informed consent forms prior to their participation in the study and were given ample time to decide on their participation in the study. They were also given the option to withdraw even after initial consent. All interviews and group discussions were held in areas with visual and auditory privacy. No personal identifiers were collected from the study respondents.

## Results

**Fig. 1:**
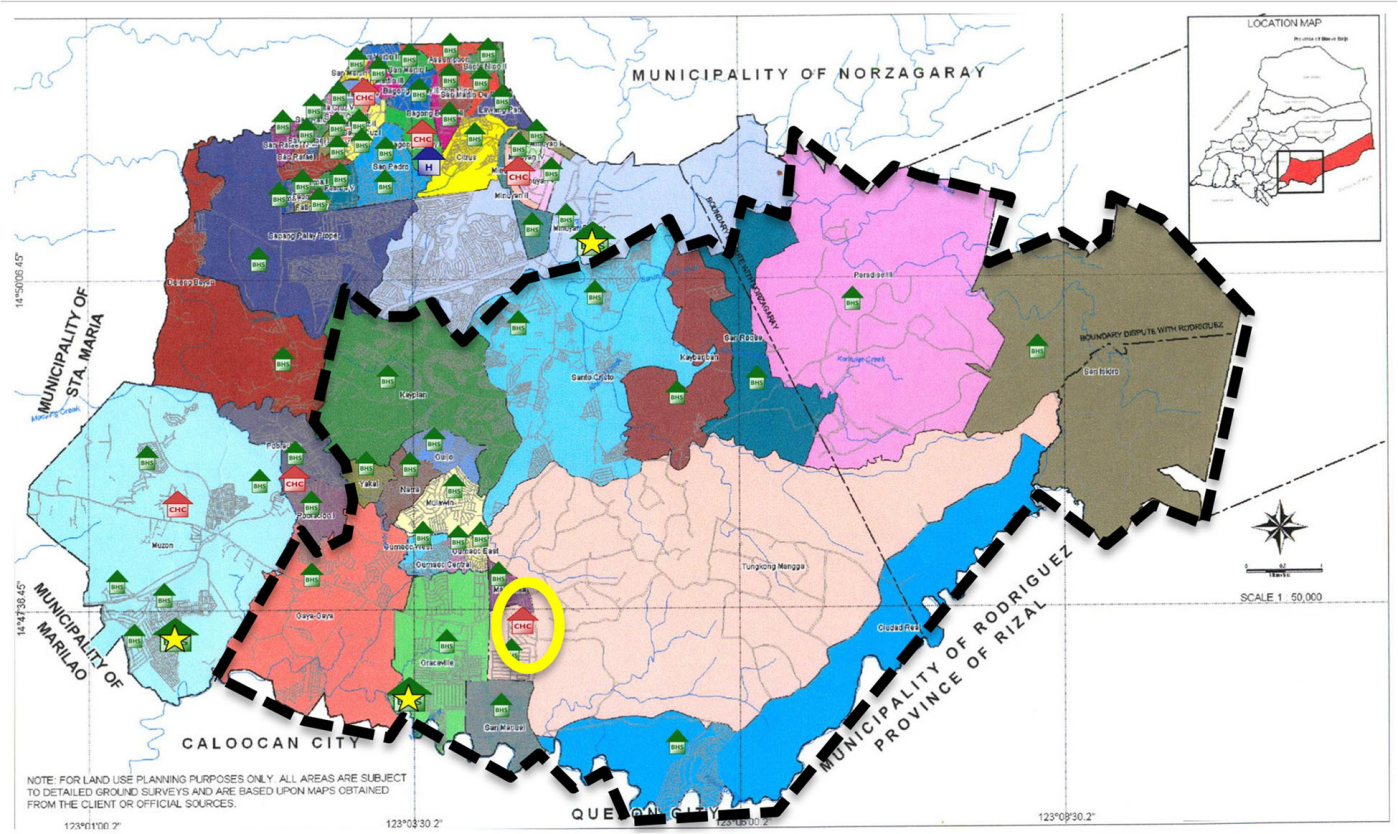
Study Site Map.  represent the Barangay (Village) Health Stations.  represent the City Health Centers.  represent the City Hospital.  represent the Barangay (Village) Health Stations with Birthing Facilities available. Map Source: City Health Office of San Jose del Monte, Bulacan, Philippines

Figure 1 shows the areas of SJDM where TB patients and BHW respondents were recruited (dashed black lines). This area has one City Health Center or CHC (encircled in yellow) where *barangay* health stations (BHS) in each village send their new TB patients for registration, HCT, and disbursement of medicines. There were 89 patients who participated in the study and had a mean age of 41.4 (SD: 15.8) while the 81 BHWs who participated had a mean age of 48.3 (SD: 8.4). Notably, more than half (68.5%) of patients were males, while almost all (96.3%) BHWs were females. Almost two-thirds (56.5%) were living less than 1 km from a *barangay* health station. Please refer to Table 1 for other characteristics of the survey respondents.

**Table 1:** Characteristics of Survey Respondents

TB patients (*n* = 89)	n	%
Male	61	68.5
Married	49	55.1
Finished High School	37	41.6
Unemployed	64	71.9
Distance of residence from the nearest *barangay* health station		
< 1 km	50	56.5
> 1 km	39	43.5
Type of TB Patient		
First-time	63	70.8
Retreatment	26	29.2
Reported being asked if they want to be tested for HIV at initial TB diagnosis	68	76.4
Had themselves HIV tested (*n* = 88)	80	89.8
Reported receiving HIV Counseling before blood extraction	54	60.7
Reported that relationship between TB and HIV was explained to them	58	53.9
Barangay Health Workers (*n* = 81)	n	%
Female	78	96.3
Married	66	81.5
Finished at least high school	65	80.3
Temporary employment status (contract renewed every year)	50	61.7
Honorarium per month (receives less than US$ 60 per month)	45	55.6

### Acceptability from patients’ perspective

The majority of TB patients (*n* = 83) preferred to receive HIV counseling from physicians while less than half (*n* = 42) of TB patients preferred to receive HIV counseling from BHWs. Patients mostly preferred (*n* = 70) to receive HIV testing from medical technologists, while only one-third (*n* = 27) agreed to receiving HIV testing from BHWs (see Table 2 for further details on patient preferences for other types of health service providers present at the other local health stations).

**Table 2:** Patients’ preferences of HCT provider (*n* = 89)

Health provider	% Agree (n) to receive HIV Counseling from this provider^a^	Health provider	% Agree (n) to receive HIV Testing from this provider^a^	*p*-value^b^
Doctor	93.3 (83)	Doctor	60.7 (54)	< 0.01
Nurse	68.2 (61)	Nurse	68.2 (61)	NS
Medical Technologists	76.5 (68)	Medical Technologists	78.8 (70)	NS
Midwife	44.2 (39)	Midwife	33.7 (30)	NS
BHW	47.2 (42)	BHW	30.3 (30)	0.02

^a^ No statistically significant difference in proportion between males and females (*χ*2 test)^b^ Z-test for two proportionsNote: Multiple responses allowed. NS = Not significant

**Table 3:** Summary of Acceptability from various stakeholders involved in the study

QUANTITATIVE	ACCEPTABILITY	
	Delegating HIV Counseling and Testing	
Barangay Health Workers	37% agree^1, a^	
QUALITATIVE^2^	ACCEPTABILITY	
	Delegating HIV counseling	Delegating HIV testing
Barangay Health Workers	Agree^2^	Disagree^2^
Other primary healthcare personnel	Agree, but only select BHWs who meet certain criteria^2^	Disagree^2^
Program managers	Agree, but only select BHWs who meet certain criteria^2^	Disagree^2^

^a^No statistically significant difference between those who received less than US$ 60 per month and those who received more than US$ per month (χ2 = .095, *p* = .758), and type of employment status either as temporary, permanent or contractual (χ2 = .158, *p* = .924), ^1^ Surveys; ^2^ FGDs

A TB patient felt that BHWs may conduct HIV counseling but suggested certain behaviors that BHWs should have so that they can be perceived as reputable sources of information:

*BHWs should act in such a way that us patients would believe them, as if they’re telling the truth… they [should] have a reference that they will read to patients… Explain to us while they’re holding a book, the MIMS maybe. So that we can be assured that what they’re saying to us is true.* (TB Patient, Female)

Results from the survey further indicate that only 33% of TB patients felt afraid that BHWs would gossip about their HIV status if BHWs were the ones to do HIV counseling and testing.

### Acceptability from BHWs’ perspective

We found that two out of three (63%) BHWs disagreed to be providers of HIV counseling and testing. However, two out of three BHWs (68%) also said that given additional training, they felt they can do community-based HIV counseling and testing for TB patients. Further probing during the FGDs suggest that BHWs felt more willing to be providers of HIV counseling rather than HIV testing.

*It’s better if just [HIV] counseling, because that is really the job of a BHW. Because we are frequent in the area, we know the households. For the HIV testing, only medical technologists have the right to do that. In case you made a mistake in pricking a patient, isn’t that illegal? Testing is not for us, we don’t even have a license.* (BHW Leader, female)

BHW leaders also felt that only select BHWs should be enlisted as providers of HIV counseling. Respondents felt that some BHWs are not committed hence it would be a waste to train all BHWs in providing TB/HIV care.

*There are BHWs for a few years now but they’re just playing around. Although there are some who really do their work well, but there are others who are just after the honorarium, and don’t know anything about the work of a BHW.* (BHW Leader, female)

### Acceptability from TB/HIV stakeholders’ and program managers’ perspective

Stakeholders were unanimous in saying that only *select* BHWs may be able to contribute to HIV counseling, group education and information dissemination campaigns, but not HIV testing. Stakeholders felt that most BHWs cannot be taught how to do HIV testing because of their low skillset, and that patients themselves might not even approach BHWs for HCT because of their reputation as gossips. Hence, stakeholders specified certain eligibility criteria for BHWs who could offer HIV counseling such as: (a) at least a high school graduate, (b) active and dedicated to BHW duties, (c) having the compassion to counsel and communicate, (d) non-judgmental attitude towards males having sex with males, sex workers, injecting drug users, and (e) having good grasp of TB/HIV.

Responding to other stakeholders’ concerns about the lack of technical capacity of BHWs to provide HIV testing, an international NGO representative from the FGDs felt that enlisting BHWs as providers might be the way to go in making HCT accessible at the community level given the resource limitations of the country in hiring more manpower. He cited the High Impact – Five program [16], the Philippine government’s initiative to provide critical interventions to achieve Universal Health Coverage among poor Filipinos, where BHWs were tasked to conduct random blood sugar testing at the community level (Table 3).

*During the High Impact-5 program last year, the Department of Health distributed glucometers nationwide, and the BHWs are doing home visits, bringing glucometer and they’re pricking people to check for their blood sugar. If the HIV rapid test is as simple as that they can do it.* (International NGO representative, male)

### Feasibility

Various feasibility requirements previously identified by stakeholders were compared with the existing conditions in SJDM (Table 4). An over-all feasibility rating (mean ± SD) of 3.12 ± 1.06 (moderate feasibility) was computed. In addition, the feasibility requirements were stratified with rating scores (mean ± SD) as follows: financial requirements (2.9 ± 0.9), infrastructural and operational requirements (3.2 ± 1.1), technical and training requirements (3.3 ± 1.1), and BHW characteristics (2.9 ± 1.1).

**Table 4:** Feasibility Requirements and Existing Conditions in SJDM Health Facilities (*N* = 17)

Financial Requirements	Existing Conditions in SJDM	Rating
1. Additional honorarium or incentives for BHWs	Χ Needs to be budgeted	3.25
2. Budget for training (approx. US$ 300 to US$ 500/trainee)	Χ Needs to be budgeted	2.5
Infrastructure and Operation Requirements	Existing Conditions in SJDM	Rating
1. Doctors available 5× a week	o 17.6% of facilities have full-time doctors	2.75
2. Rooms with visual and/or auditory privacy	o 35.3% of facilities have rooms with visual and auditory privacy	2.75
3. Mechanism for infectious waste disposal	✓ Existing	3.75
4. Guidelines/responsibilities of BHWs as HCT providers	Χ Not yet existing	2.75
5. Timely delivery of medical supplies	o Supply chain mechanisms take 1 to 2 months before delivering supplies	3.25
6. Delineate deaths caused by TB alone and deaths caused by TB/HIV	✓ Can be extrapolated from existing TB and HIV databases	3.5
7. Agency to monitor the implementation of BHWs as HCT providers	✓ TB/HIV collaboration group existing	2.75
8. Establishing linkages with NGOs	✓ SJDM has limited partnerships with NGOs	3.75
Technical and training requirements	Existing Conditions in SJDM	Rating
1. Training on basic TB/HIV knowledge	✓ 88.2% of BHWs trained on basic HIV knowledge; 23.5% trained on TB/HIV in the last two years;	3
2. Training on how to use the HIV rapid test kits	✓ 5.9% of BHWs in facilities trained on HCT	2
3. HIV awareness campaigns in the community	✓ SJDM stakeholders conducted community awareness campaigns	4
4. HCT refresher course for other primary health center personnel	✓ Physicians and nurses trained on HCT in the last two years; less than 10% of midwives in facilities trained in HCT	3.75
5. Knowledge transfer mechanisms from upper to lower levels of the health system	o Capacity building activities available but knowledge transfer mechanisms vague	3.75
BHW Characteristics	Existing Conditions in SJDM	Rating
1. Time spent for BHW responsibilities less than working time per week	✓ Working minutes per week: 1920 mins; time spent for responsibilities: 1575 mins	2.75
2. BHWs are high school graduates	✓ 71% of BHWs are high school graduates	3.25
3. Few stigmatization behaviors towards TB/HIV patients among BHWs	✓ 20.3% of BHWs have hesitations in talking to a patient with TB/HIV	2.75
4. Good knowledge in patient counselling principles among BHWs	o Only 16.5% have knowledge; 69.1% needs refresher course	3

Legend: ✓ - Criteria fulfilled; ο – Criteria partially fulfilled; X – Criteria not fulfilled

(Table 4. Feasibility Requirements and Existing Conditions in SJDM Health Facilities)

## Discussion

Our study is one of the few to provide evidence of the feasibility and acceptability of delegating HIV counseling to CHWs from an Asian country with a devolved health system and concentrated HIV epidemic. In contrast, other studies have been done in sub-Saharan Africa and India where CHWs are already tasked to provide TB and HIV care at the primary care level [7, 11, 17]. Our study added to the evidence of the acceptability of HCT if CHWs were to offer it, in contrast to other studies that examined its acceptability if healthcare professionals would offer it [18, 19]. We were also able to provide the feasibility requirements inductively based on actual implementation conditions and not just as a function of how many TB patients were referred for HIV testing as done by other studies [17, 20].

This study aimed to provide evidence that patients would not need to travel to the City Health Centers to access health care, instead, patients could receive their medications, and HCT from the nearest BHS, echoing the principles of patient-centered TB care [21]. However, we were able to find that in San Jose del Monte City, delegation of both HIV counseling and testing to BHWs is moderately feasible but only delegation of HIV counseling is acceptable. Stakeholders were unanimous in saying that select BHWs who possess certain characteristics can provide HIV counseling services but not HIV testing.

That respondents perceived it not acceptable for BHWs to test may be because of the confidentiality clauses specified by the Philippine AIDS Prevention and Control Act of 1994, the Philippine Medical Technology Act of 1969, and Administrative Order 2010–0028 of the Department of Health (DOH) which exclusively limited the collection and examination of hematologic samples, and HIV *testing* to medical technologists who are certified as HIV-proficient [22–24]. It can also be gleaned that the lack of acceptability maybe because BHWs are yet to be seen as reliable sources of information (perspective of TB patients) and only a select few are perceived to have the technical skills to do HIV testing (perspective of program managers). It is interesting to note that while program managers mentioned about BHWs being gossips, we found that only 1 in 3 patients were afraid that BHWs would gossip about their HIV status. It seems then that community members may be open to the idea of receiving HIV testing from BHWs in the near future, provided BHWs establish themselves as credible and knowledgeable service providers. Indeed, BHWs themselves disagree to become HCT providers at present because they felt that they needed to have more training. Mechanisms that ensure BHW-led HCT services are delivered professionally and safeguards that protect patient confidentiality should thus be created.

Paradoxically, however, other trained lay providers from community-based MSMs and transgender peoples’ organizations are already conducting community-based HIV *screening* initiatives sanctioned by the Philippines’ Department of Health. These initiatives are called *screening* because they are not considered as ‘official’ HIV testing (i.e. if reactive during HIV screening initiatives, they will be referred to HIV testing facilities for HIV testing from a HIV-proficient medical technologist). While this could be done San Jose del Monte, it may pose some problems since there are only two medical technologists and two physicians stationed in the city center catering to around 300,000 people. Hence, there may be a need to evaluate the Philippine national policies on HCT to accommodate lay HCT providers to include barangay health workers. Further studies are also needed given WHO recommendations and other studies which found that HCT services provided by CHWs are at par with those by trained healthcare providers [25–28].

Given the acceptability of delegating HIV counseling and group education to BHWs, it might then be wise to engage them as agents to reduce stigma towards PLHIV at the community level as in Kenya [29]. Stigmatization from the general population has been reported as a significant barrier at the different levels of the HIV treatment cascade among Filipino MSMs [30, 31].

The feasibility ratings given by program managers also suggest that it is in the areas of financial requirements and BHW training where resources should be allocated. Lobbying with local government decision-makers to fund the financial support needed to capacitate BHWs and build the infrastructural requirements is needed. Given BHWs’ reputation as people who gossip (although patients do not seem to worry about this), additional soft-skills trainings on patient sensitivity and confidentiality protection may also be conducted in addition to technical trainings related to provision of TB/HIV care. In addition, health education campaigns geared towards promoting BHWs as providers of HIV care, and reducing stigma among TB and HIV patients may be necessary.

At the system level, there may also be a need to introduce measures to strengthen the Philippines’ adherence to the three recommendations for collaborative TB/HIV activities specified by the World Health Organization [32]. While the Philippines has done significant advancements in the second component (reduce the burden of TB in people living with HIV) and third component (reduce the burden of HIV in patients with presumptive and diagnosed TB), there is still a need to improve activities in the first component (establish and strengthen the mechanisms for delivering integrated TB and HIV services). In particular, there is a need to ensure that there is a functional TB/HIV coordinating body in all levels of the health system, and that TB and HIV programs in each level closely coordinate with each other in terms of monitoring service delivery. Both of these however, pose a challenge because of the devolved setup of the Philippine health system. Strengthening the TB/HIV collaboration mechanisms especially at the primary healthcare level is important as it will serve as the backbone which will legitimize BHWs as providers of TB and HIV care.

### Study limitations

Our study is similar to an in-depth case study, which triangulated multiple sources of data to answer its research questions. While the study elicited the opinion of the medical technologists in the study area, it may have been better to have elicited their opinions as a group separate from other primary healthcare personnel. We believe, however, that this will not affect the over-all validity of the findings because medical technologists are only involved in drawing blood specimens and HIV testing; the programmatic decision-making is still with program managers and physicians, the opinions of both have been rigorously examined in the study. As with any survey, results are subject to social desirability bias and these were handled through the use of triangulation with qualitative data, and validation with other stakeholders. Future qualitative studies should also explore TB patients’ preferences for testing and why they do not prefer to receive HIV testing from BHWs.

The findings of the study may only be generalized in other health systems where local government executives exercise high degree of autonomy over health priorities, a high TB and HIV burden occurs, where there are huge discrepancies of HCT provider-to-population ratios, and where topography is characterized by a mix of urban and rural areas, plains and mountainous villages.

## Conclusion

The study found that in the Philippines delegation of both HIV counseling and testing is moderately feasible, but only delegation of HIV counseling to select BHWs who pass certain criteria is acceptable. Further studies are needed to increase the acceptability of delegating HCT to Filipino BHWs in order to mainstream the delegation of tasking BHWs to deliver HIV counseling. There is also a need to establish and strengthen mechanisms for delivering integrated TB and HIV services especially at the primary healthcare level.

## Additional file

Additional file 1: QUALITATIVE INSTRUMENT 1: FGD FOR BARANGAY HEALTH WORKERS or BHWs (ENGLISH VERSION). QUALITATIVE INSTRUMENT 2: Focus Group Discussion Guide for Program Managers, Decision-makers and Key Stakeholders. QUALITATIVE INSTRUMENT 3: Focus Group Discussion Guide for Primary Healthcare Personnel. Exhibit A. TB/HIV Treatment Flowchart in Zambia. Exhibit B. Programmatic Implementation of TB/HIV Care in India. Qualitative Instrument 4: Semi-structured Interview Guide for TB Patients Post-Survey Interview. QUANTITATIVE INSTRUMENT 1: KAPs of BHWs. Perceptions of Appropriateness of Delegating HIV testing among TB to CHWs. QUANTITAT QUANTITATIVE INSTRUMENT 3: SURVEY FOR TB PATIENTSIVE INSTRUMENT 2: FACILITY ASSESSMENT. QUANTITATIVE INSTRUMENT 4: FEASIBILITY RATING OF PROGRAM MANAGERS. (DOCX 6891 kb)

## Abbreviations

ART: Anti-retroviral Therapy
BHS: Barangay Health Stations
BHW: Barangay Health Workers
CHC: City Health Center
CHW: Community Health Workers
DOH: Department of Health (Philippines)
HCT: HIV Counseling and Testing
HIV: Human Immunodeficiency Virus
MSM: Males having sex with Males
NGO: Non-government Organizations
PITC: Provider Initiated Testing and Counseling
SJDM: San Jose del Monte
TB: Tuberculosis
WHO: World Health Organization
