# Supplementary material for: Acceptability and feasibility of delegating HIV counseling and testing for TB patients to community health workers in the Philippines: a mixed methods study
Source: BMC Public Health. 2019 Feb 13;19:185. doi: 10.1186/s12889-019-6497-7 (PMC6375216; doi:10.1186/s12889-019-6497-7)
Supplement: Supplementary file 1 — QUALITATIVE INSTRUMENT 1: FGD FOR BARANGAY HEALTH WORKERS or BHWs (ENGLISH VERSION). QUALITATIVE INSTRUMENT 2: Focus Group Discussion Guide for Program Managers, Decision-makers and Key Stakeholders. QUALITATIVE INSTRUMENT 3: Focus Group Discussion Guide for Primary Healthcare Personnel. Exhibit A. TB/HIV Treatment Flowchart in Zambia. Exhibit B. Programmatic Implementation of TB/HIV Care in India. Qualitative Instrument 4: Semi-structured Interview Guide for TB Patients Post-Survey Interview. QUANTITATIVE INSTRUMENT 1: KAPs of BHWs. Perceptions of Appropriateness of Delegating HIV testing among TB to CHWs. QUANTITAT QUANTITATIVE INSTRUMENT 3: SURVEY FOR TB PATIENTSIVE INSTRUMENT 2: FACILITY ASSESSMENT. QUANTITATIVE INSTRUMENT 4: FEASIBILITY RATING OF PROGRAM MANAGERS. (DOCX 6891 kb) [file 12889_2019_6497_MOESM1_ESM.docx]

**QUALITATIVE INSTRUMENT 1 FGD FOR BARANGAY HEALTH WORKERS or BHWs (ENGLISH VERSION)**

**Focus group discussion guide:**

1. Please describe to me about your daily work life as BHWs, how your day usually begins, what your duties are and what you do?
   1. Here in San Jose del Monte, how do BHWs support the staff of the health center?
   2. How about the roles that you do in the Community/Village?
2. What is the most difficult part of your job as a BHW?
   1. What keeps you going in this line of work?
   2. Any work-related challenges that you face, security of tenure, political issues, etc?
3. Tell me about your work in tuberculosis diagnosis, treatment, surveillance here in San Jose del Monte.
   1. Any patient counseling and testing activities related to TB?
4. Are you familiar with the connection between TB and HIV?
   1. Tell me more about what you know about this connection.
   2. Any training activities being conducted related to TB/HIV care so far?
   3. What is the role of BHWs here in San Jose del Monte with regard to HIV testing among TB patients?
5. Ideally, HIV patients should be tested for TB, and TB patients should be tested for HIV. Are you familiar with this idea?
6. We currently have a problem with regard to uptake of HIV testing among TB patients, and one problem is that there are insufficient number of medical technologists certified to do this especially at the primary health care level. One of the solutions to this is to delegate HIV testing among TB patients to BHWs as is being done in Africa and India.
   1. How do you feel about it if the same system would also be done here in the Philippines?
   2. Is this appropriate?
   3. Is this acceptable?
7. Here are some of the ways in which TB/HIV care are integrated in other countries. Show Exhibit A and Exhibit B to respondents (next page)
   1. Do you think this can be done in the Philippines? Here in San Jose del Monte?
   2. Where do you think BHWs can come in, in these treatment flowcharts?
   3. What do you think are the contributions that you can provide as a BHW to HIV testing for TB patients and v.v.?
8. Considering what you have here now in the community, what other things do you think are still needed if HIV testing for TB patients would be delegated to BHWs?
   1. Training needed?
   2. Logistics?
   3. Infrastructure needed?
   4. With right training and all the equipment needed, would you be confident to conduct HIV counseling and testing to TB patients? Why or why not?
9. One of the interventions that government is introducing now is HIV Self-Testing
   1. Do you think BHWs should be tapped as human resource complements in this new intervention? Why or why not?
   2. If yes, what do you think are the contributions that BHWs can provide to its implementation?

**QUALITATIVE INSTRUMENT 2: Focus Group Discussion Guide for Program Managers, Decision-makers and Key Stakeholders**

1. (Introduction)
2. We are already a bit familiar with the burden of TB and HIV, separately, but how about TB/HIV co-infection?
   1. What are proposed/existing government interventions being done on TB/HIV co-infection?
   2. How does the government and the DOH perceive TB/HIV co-infection as a health issue? (i.e. is it a priority, is it something considered urgent?)
3. In your opinion, how is the integration of TB/HIV care in the Philippines/the province/district?
   1. What are the barriers and enablers to the integration of TB/HIV care in the Philippines/your province/your district?
      1. Which of these factors affect TB/HIV integration in Philippines/your province/your district? poor knowledge about TB/HIV, lack of communication skills and knowledge among HIV service providers, insufficient financial support, absence of target plan of TB patients referred for HIV testing, fear of knowing test result/stigma among TB patients, strategies and guidelines not in place, limited VCT centers, poor commitments from health staff, poor collaboration between TB and HIV/AIDS program staff
   2. How about challenges to implementation, especially with regard to HIV testing among TB patients? And the implementation issues regarding shared roles prescribed by 2014-0005?
   3. Are there any organizational culture differences between TB and HIV programs? If yes, do you think this could influence how you collaborate/integrate TB/HIV care?
      1. Probe: TB as more technical or medicine-based, and HIV as more human-rights based
4. We currently have some challenges with regard to uptake of HIV testing among TB patients, and one problem cited is that there are insufficient number of medical technologists certified to do this especially at the primary health care level.
   1. Do you feel that this a valid explanation to the uptake issue? Any other thoughts/potential explanations regarding this?
5. One of the solutions to this lack of HRH at the primary health care level is to delegate HIV testing among TB patients to BHWs as is being done in Africa and India.
   1. How do you feel about it if the same system would also be done here in the Philippines?
   2. Is this appropriate?
   3. Is this acceptable?
6. Here are some of the ways in which TB/HIV care are integrated in other countries. Show Exhibit A and Exhibit B to respondents (next page)
   1. Do you think this can be done in the Philippines? Especially in Category A areas like San Jose del Monte City, Bulacan?
   2. Where do you think BHWs can come in, in these treatment flowcharts?
   3. What do you think are the contributions that you can provide as a BHW to HIV testing for TB patients and v.v.?
7. Considering our local context and resources, what other things do you think are still needed if HIV testing for TB patients would be delegated to BHWs?
   1. Training needed?
   2. Logistics and financial requirements needed?
   3. Infrastructure needed?
8. What are the areas that you think still need to be done, especially with regard to optimizing the provision of TB/HIV care in the country?
   1. For NGO key stakeholders: potential contributions/roles that you can provide as organizations concerned with TB/HIV?

*Wrap-up FGD*

1. One of the interventions that government is introducing now is HIV Self-Testing
   1. TO Provincial/District respondents: Is this appropriate and/or acceptable?
   2. What should be the role of primary healthcare center personnel in the potential implementation of this intervention?
   3. Do you think BHWs should be tapped as human resource complements in this new intervention? Why or why not?
   4. If yes, what do you think are the contributions that BHWs can provide to its implementation?

**QUALITATIVE INSTRUMENT 3: Focus Group Discussion Guide for Primary Healthcare Personnel**

1. We are already a bit familiar with the burden of TB and HIV, separately, but how about TB/HIV co-infection especially in your locality?
   1. What are proposed/existing local government interventions being done on TB/HIV co-infection?
   2. How does the local government and the regional/provincial DOH perceive TB/HIV co-infection as a health issue? (i.e. is it a priority, is it something considered urgent?)
2. In your opinion, how is the integration of TB/HIV care here in your locality?
   1. What are the barriers and enablers to the integration of TB/HIV care?
      1. Which of these factors affect TB/HIV integration in your locality? Poor knowledge about TB/HIV, lack of communication skills and knowledge among HIV service providers, insufficient financial support, absence of target plan of TB patients referred for HIV testing, fear of knowing test result/stigma among TB patients, strategies and guidelines not in place, limited VCT centers, poor commitments from health staff, poor collaboration between TB and HIV/AIDS program staff
   2. How about challenges to implementation, especially with regard to HIV testing among TB patients? And the implementation issues regarding shared roles prescribed by 2014-0005?
3. We currently have some challenges with regard to uptake of HIV testing among TB patients, and one problem cited is that there are insufficient number of medical technologists certified to do this especially at the primary health care level.
   1. Is this a valid explanation to the uptake issue, especially here in your locality? Any other thoughts/potential explanations regarding this?
4. One of the solutions to this lack of HRH at the primary health care level is to delegate HIV testing among TB patients to BHWs as is being done in Africa and India.
   1. What is the current role of BHWs in provision of HIV testing among TB patients here in your locality?
   2. How do you feel about it if the same system would also be done here in the Philippines?
   3. Is this appropriate?
   4. Is this acceptable?
5. Here are some of the ways in which TB/HIV care are integrated in other countries. Show Exhibit A and Exhibit B to respondents (next page)
   1. Do you think this can be done especially in Category A areas like San Jose del Monte City, Bulacan?
   2. Where do you think BHWs can come in (i.e. contributions they can give), in these treatment flowcharts especially to support primary healthcare center personnel if this intervention gets implemented?
6. Considering your existing context and resources, what other things do you think are still needed if HIV testing for TB patients would be delegated to BHWs?
   1. Training needed?
   2. Logistics and financial requirements needed?
   3. Infrastructure needed?
7. One of the interventions that government is introducing now is HIV Self-Testing
   1. Is this appropriate and/or acceptable?
   2. What should be the role of primary healthcare center personnel in the potential implementation of this intervention?
   3. Do you think BHWs should be tapped as human resource complements in this new intervention? Why or why not?
   4. If yes, what do you think are the contributions that BHWs can provide to its implementation?

**EXHIBIT A and B**

**Exhibit A. TB/HIV Treatment Flowchart in Zambia**


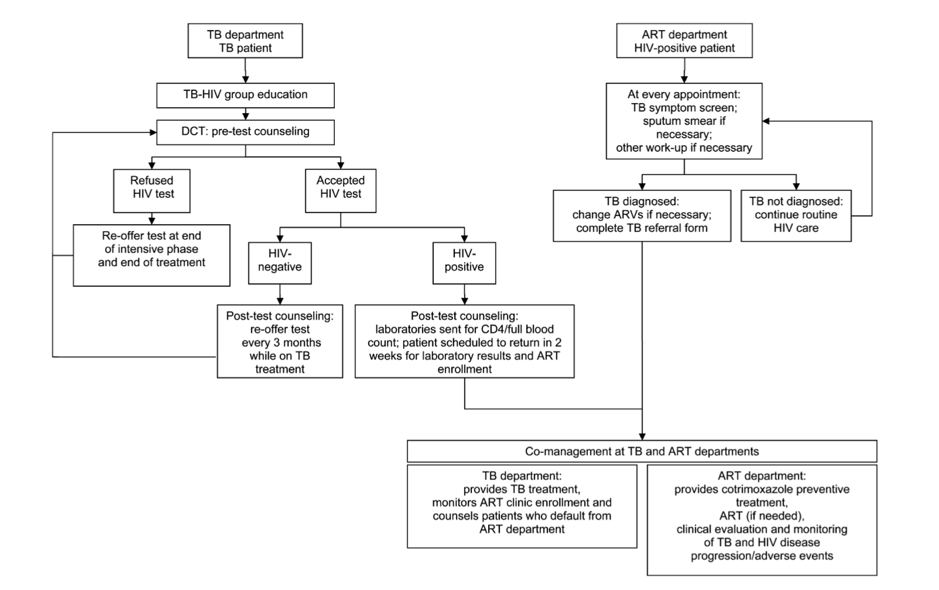


Taken from J. B. Harris, S. M. Hatwiinda, K. M. Randels, B. H. Chi, N. G. Kancheya, M. A. Jham, K. V. G. Samungole, B. C. Tambatamba, R. A. Cantrell, J. W. Levy, M. E. Kimerling, S. E. Reid (2008). Early lessons from the integration of tuberculosis and HIV services in primary care centers in Lusaka, Zambia. INT J TUBERC LUNG DIS 12(7):773–779.

**Exhibit B. Programmatic Implementation of TB/HIV Care in India**


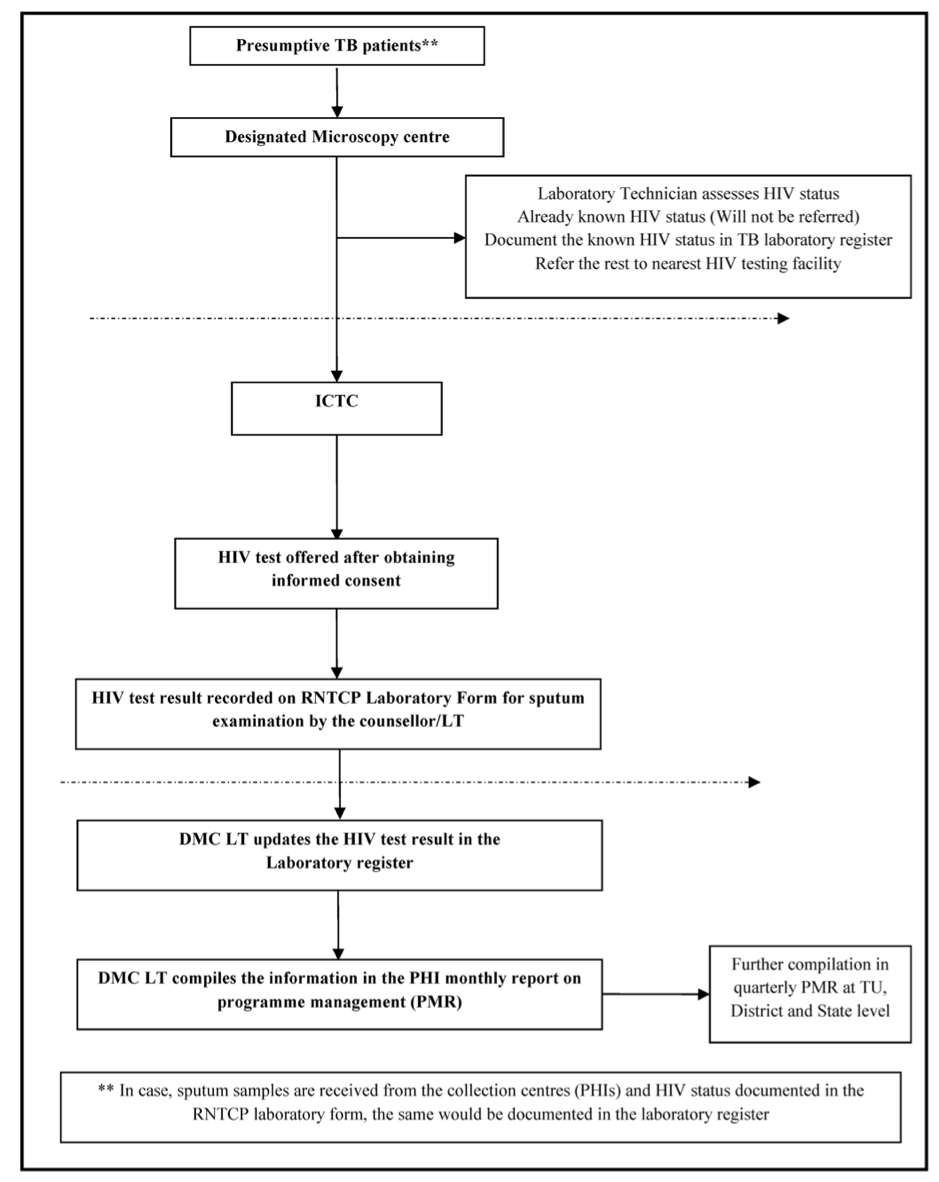


Taken from: Kumar AMV, Gupta D, Kumar A, Gupta RS, Kanchar A, Rao R, et al. (2016) HIV Testing among Patients with Presumptive Tuberculosis: How Do We Implement in a Routine Programmatic Setting? Results of a Large Operational Research from India. PLoS ONE 11(5): e0156487. doi:10.1371/journal. pone.0156487

**Qualitative Instrument 4: Semi-structured Interview Guide for TB Patients Post-Survey Interview**

1. (Introduction, Names, Icebreaker/Light Questions)
2. What do you know about HIV or AIDS? What do you know about the relationship between TB and HIV? Can you describe it to me please?
3. As a TB patient, do you know that you need to be tested for HIV?
4. Would you be willing to get tested for HIV?
   1. Why or why not?
   2. Probe whether these factors influence decision: anonymity, stigma, lack of information, lack of social support, lack of perceived risk, fears of testing positive
5. Where would you want to get tested for HIV?
   1. What are the factors that you will consider in a place where you would want to be tested?
   2. Probe whether these factors influence choice of testing site: distance of testing center from house, physical characteristics of testing center, price of testing, type of facility, previous experiences being tested
6. Who would you want to conduct the HIV counseling and testing to you?
   1. Probe: physician, nurse, or maybe not necessarily physician/nurse but a provider in white coat?
   2. Probe: physical and emotional/personality characteristics of preferred HIV counselor
7. If BHWs would be the one to conduct HIV testing, would you avail of their services? Why or why not?
   1. What are the characteristics of a BHW that would **encourage** you to avail of HIV counseling and testing from them?
   2. What are the characteristics of a BHW that would **discourage** you to avail of HIV counseling and testing from them?
   3. What are your hesitations, fears, or dilemmas in availing of HIV Counseling and testing from BHWs?
8. (Explain self-testing first; show a self-testing kit). One of the government’s proposed interventions is HIV self-testing.
   1. What are your thoughts or opinions about this intervention?
   2. Is this a better or worse alternative to having yourself tested at HIV testing centers or at the health centers?
   3. If ever this intervention gets implemented, what do you think should be the roles of BHW and primary healthcare center personnel to support patients who want to avail it?

**QUANTITATIVE INSTRUMENT 1: KAPs of BHWs**

Greetings! We would like to ask 15 to 30 minutes of your time to answer the following questions. There are no right or wrong answers and we would appreciate your honesty in answering this questionnaire. **Please note that this will *NOT* serve as an evaluation of your work performance as a BHW.** Hence, it would really help us if you answer all the questions however, please feel free to leave any questions you do not want to answer. If you have any questions, please do not hesitate to contact the person who gave this questionnaire to you. Thank you!

**Respondents’ Demographic Information**

[Q1.1] Age: _____

[Q1.2] Sex: [ ] Male [ ] Female

[Q1.3] Civil Status:

[ ] Single

[ ] Married

[ ] Live-in

[ ] Widowed

[ ] Separated

[Q1.4] Highest Educational Attainment:

[ ] No Formal Schooling

[ ] Finished pre-school (kinder, pre-elementary)

[ ] Finished Elementary

[ ] Finished High School

[ ] Finished College

[ ] Finished Postgraduate Studies

[ ] Vocational/Technical

[Q1.6] Number of MONTHS in service as BHW: _______

[Q1.7] Employment Status as BHW:

[ ] Permanent

[ ] Contractual

[ ] Voluntary

[Q1.8] Honorarium per month received as BHW:

[ ] PhP 3,000 above

[ ] PhP 1,001 to 2,999

[ ] PhP 600 to 1,000

[ ] PhP 401 to 599

[ ] PhP 201 to 400

[ ] PhP 200 and below

[ ] Receives no honorarium

[Q1.9] Monthly Family Income (all breadwinners in the family)

[ ] PhP 1,000 – 5,000

[ ] PhP 5,001 – 10,000

[ ] PhP 10,001 – 15,000

[ ] PhP 15,001 – 20,000

[ ] PhP 20,001 and above

**Perceptions of Appropriateness of Delegating HIV testing among TB to CHWs**

Below are series of statements regarding delegation of HIV testing among TB patients to CHWs. Put an **X** in the column which accurately represents your agreement to the statement.

|  | 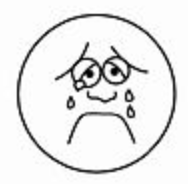  Very Strongly  Disagree | 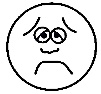  Strongly Disagree | 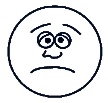  Disagree | 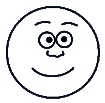  Agree | 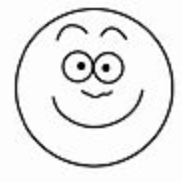  Strongly  Agree | 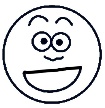  Very Strongly Agree |
| --- | --- | --- | --- | --- | --- | --- |
| 1. In my opinion, BHWs should be the ones to provide community-based HIV counseling and testing to TB patients. |  |  |  |  |  |  |
| 1. In my opinion, only **doctors** at the TB-DOTS unit should do community-based HIV counseling and testing for TB patients, not BHWs. |  |  |  |  |  |  |
| 1. In my opinion, only **nurses** at the TB-DOTS unit should do community-based HIV counseling and testing for TB patients, not BHWs. |  |  |  |  |  |  |
| 1. In my opinion, only **midwives** at the TB-DOTS unit should do community-based HIV counseling and testing for TB patients, not BHWs. |  |  |  |  |  |  |
| 1. Only staff of the social hygiene clinic should do HIV counseling and testing among TB patients. |  |  |  |  |  |  |
| 1. In my opinion, delegating HIV testing among TB patients to BHWs would only lead increased work burden. |  |  |  |  |  |  |
| 1. Assessment of HIV risk factors in TB patients can only be done by either doctors, nurses, or midwives in the TB-DOTS unit. |  |  |  |  |  |  |
| 1. Patient referral to HIV unit can only be done by either doctors, nurses, or midwives in the TB-DOTS unit. |  |  |  |  |  |  |
| 1. BHWs should be the ones to provide community-based HIV counseling and testing among TB patients because they are more knwoledgeable about their community’s residents. |  |  |  |  |  |  |
| 1. I feel motivated to conduct community-based HIV counseling and testing among TB patients. |  |  |  |  |  |  |
| 1. I feel I have enough training and skills to provide community-based HIV counseling and testing to TB patients. |  |  |  |  |  |  |
| 1. Given additional training, I feel I can do community-based HIV counseling and testing for TB patients. |  |  |  |  |  |  |
| 1. BHWs can guide TB patients to do HIV self-testing. |  |  |  |  |  |  |
| 1. BHWs can greatly contribute to the implementation of HIV self-testing in communities. |  |  |  |  |  |  |

**Workload Assessment**

On what days do you usually perform your duties as a BHW? Please check

[ ] Monday [ ] Tuesday [ ] Wednesday [ ] Thursday

[ ] Friday [ ] Saturday [ ] Sunday

On average, what time do you usually START your duties: ____________

On average, what time do you usually FINISH your duties: ____________

Which of these activities do you perform each week? Put an **X** to mark your choices.

| **DUTY** | **X** | **DUTY** | **X** | **DUTY** | **X** |
| --- | --- | --- | --- | --- | --- |
| 1. Giving treatment to simple diseases such as fever, colds and cough |  | 1. Identifying community health needs & problems |  | 1. Maintaining linkages and referral systems |  |
| 1. Active case finding for disease cases (e.g. TB, dengue, diarrhea) |  | 1. Keeping records of work and health activities |  | 1. Conduct household teachings on personal hygiene |  |
| 1. Motivating mothers for breastfeeding |  | 1. Assisting community members |  | 1. Conduct household teachings on sanitation |  |
| 1. Updating growth monitoring cards |  | 1. Conducting household and family surveys |  | 1. Others (pls specify): |  |
| 1. Immunization campaign |  | 1. Maintaining regular communication with community leaders and professional health workers |  | 1. Others (pls specify): |  |

Approximately how many hours do you spend PER WEEK on each of these activities? **Kindly answer for all those you have marked above.**

| **DUTY** | **HR** | **DUTY** | **HR** | **DUTY** | **HR** |
| --- | --- | --- | --- | --- | --- |
| 1. Giving treatment to simple diseases such as fever, colds and cough |  | 1. Identifying community health needs & problems |  | 1. Maintaining linkages and referral systems |  |
| 1. Active case finding for disease cases (e.g. TB, dengue, diarrhea) |  | 1. Keeping records of work and health activities |  | 1. Conduct household teachings on personal hygiene |  |
| 1. Motivating mothers for breastfeeding |  | 1. Assisting community members |  | 1. Conduct household teachings on sanitation |  |
| 1. Updating growth monitoring cards |  | 1. Conducting household and family surveys |  | 1. Others (pls specify): |  |
| 1. Immunization campaign |  | 1. Maintaining regular communication with community leaders and professional health workers |  | 1. Others (pls specify): |  |

**Counseling Skills**

Below are series of statements regarding the principles of HIV counseling. Put an **X** in the column which accurately represents your agreement to the statement. There are no right or wrong answers.

|  | 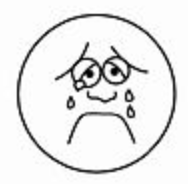  Very Strongly  Disagree | 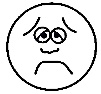  Strongly Disagree | 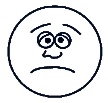  Disagree | 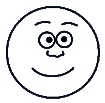  Agree | 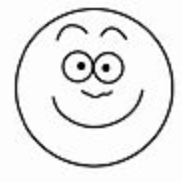  Strongly  Agree | 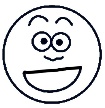  Very Strongly Agree |
| --- | --- | --- | --- | --- | --- | --- |
| 1. When doing HIV counseling, there is a need to explore the client’s knowledge, efforts or motivation. |  |  |  |  |  |  |
| 1. When doing HIV counseling, there is a need to prescribe the reasons for change or motivation to the client. |  |  |  |  |  |  |
| 1. There is a need for the HIV counselor to become an expert during the interaction with the client. |  |  |  |  |  |  |
| 1. The HIV counselor needs to collaborate and enable the client to determine his own needs independently. |  |  |  |  |  |  |
| 1. The HIV counselor needs to make the client realize that he has the potential to act towards health. |  |  |  |  |  |  |
| 1. The client should be the one to completely direct the flow of the HIV counseling session. |  |  |  |  |  |  |
| 1. HIV counselors should only focus on what the client explicitly says. |  |  |  |  |  |  |
| 1. HIV counselors should persevere to get a deeper understanding of the client’s point of view of his health experiences. |  |  |  |  |  |  |
| 1. **Open-ended questions** should be posed to clients during HIV counseling and testing. |  |  |  |  |  |  |
| 1. **Closed-ended questions** should be posed to clients during HIV counseling and testing. |  |  |  |  |  |  |
| 1. In HIV counseling, there is a need to reflect the client’s statements back to him/her. |  |  |  |  |  |  |
| 1. In HIV counseling, there is a need to ask for permission before giving advice or information to the client. |  |  |  |  |  |  |
| 1. In HIV counseling, giving orders or commands to the client is considered appropriate. |  |  |  |  |  |  |

**QUANTITATIVE INSTRUMENT 2: FACILITY ASSESSMENT**

**Note to respondent:** There are no right or wrong answers. Please be as honest as you can in answering the questionnaire as this is meant to identify potential areas of recommendation at the policy and program level. Your facility will **not** be identified individually. Data will be analyzed collectively.

[Q1] Name of Facility: __________________________________________

[Q2] Location of Facility (name of barangay): _____________________________________

[Q3] Type of Facility:

[ ] District/Provincial Hospital

[ ] City health unit/health center

[ ] Barangay health station

[ ] Lying-in clinic

[ ] Social Hygiene clinic

Is there an area or private room with…

[ ] Auditory Privacy Only

[ ] Visual Privacy Only

[ ] Both Auditory and Visual Privacy

[ ] No room/area available with both auditory and visual privacy

How many rooms with…

_____ Auditory Privacy Only

_____ Visual Privacy Only

_____ Both Auditory and Visual Privacy

[ ] Others: ________________________________

[Q4] Outpatient only? [ ] Yes [ ] No

[Q5] On average, how many hours per day is this facility open?

[ ] 4 hours or less

[ ] 5 to 8 hours

[ ] 9 to 16 hours

[ ] 17 to 23 hours

[ ] 24 hours

[Q5.1] On which days is this facility open?

[ ] Monday

[ ] Tuesday

[ ] Wednesday

[ ] Thursday

[ ] Friday

[ ] Saturday

[ ] Sunday

[Q6] Which of these health services do you offer in this facility?

[ ] Reproductive, Maternal and Newborn Health Services

[ ] Antenatal Care Services

[ ] Prevention of Mother-to-Child Transmission Services

[ ] Obstetric and Newborn Care Services

[ ] Cesarean Section

[ ] Child Immunization Services

[ ] Child Preventative and Curative Services

[ ] Adolescent Health Services

[ ] HIV Counselling and Testing

[ ] HIV Treatment

[ ] HIV Care and Support

[ ] Sexually Transmitted Infections

[ ] Tuberculosis

[ ] Malaria

[ ] Non-communicable Diseases

[ ] Surgical Services

[ ] Blood Transfusion

[ ] Diagnostics

[ ] Medicine Dispensing

[Q7] Which of the following TB/HIV services are currently being provided the health facility?

|  | Yes | No |
| --- | --- | --- |
| [Q7a] Provide HIV counselling and testing services to HIV positive pregnant women for PMTCT |  |  |
| [Q7b] Provide HIV counselling and testing services to infants born to HIV positive pregnant women for PMTCT |  |  |
| [Q7c] Provide ARV prophylaxis to HIV positive pregnant women for PMTCT |  |  |
| [Q7d] Provide ARV prophylaxis to newborns of HIV positive pregnant women for PMTCT |  |  |
| [Q7e] Provide infant and young child feeding counselling for PMTCT |  |  |
| [Q7f] Provide nutritional counselling for HIV positive pregnant women and their infants for PMTCT |  |  |
| [Q7g] Provide HIV counselling and testing services to HIV positive pregnant women for PMTCT |  |  |
| [Q7h] Provide HIV counselling and testing services to infants born to HIV positive pregnant women for PMTCT |  |  |
| [Q7i] Provide HIV counseling and testing services to adult populations |  |  |
| [Q7j] Provide HIV counselling and testing servces to minor adolescents |  |  |
| [Q7k] Provide HIV & AIDS ARV prescription |  |  |
| [Q7L] Provide ARV treatment and follow-up services |  |  |
| [Q7m] Provide HIV/AIDS care and support services, including treatment of opportunistic infections and provisions of palliative care |  |  |
| [Q7n] Prescribe treatment for any opportunitistic infections or symptoms related to HIV/AIDS including topical fungal infections |  |  |
| [Q7o] Provide or prescribe palliative care for patients, such as symptom or pain management, or nursing care for the terminally ill, or severely debilitated clients |  |  |
| [Q7p] Provide systemic intravenous treatment of specific fungal infections such as cryptococcal meningitis |  |  |
| [Q7q] Provide treatment for Kaposi's sarcoma |  |  |
| [Q7r] Provide nutritional rehabilitation services? e.g., client education and provision of nutritional supplements |  |  |
| [Q7s] Prescribe or provide fortified protein supplementation (FPS) |  |  |
| [Q7t] Care for paediatric HIV/AIDS patients? |  |  |
| [Q7u] Prescribe or provide preventive treatment for TB (INH + Pyridoxine) |  |  |
| [Q7v] Primary preventive treatment for opportunistic infections, such as co- trimoxazole preventive treatment (CPT) |  |  |
| [Q7w] Provide or prescribe micronutrient supplementation, such as vitamins or iron |  |  |
| [Q7x] Provide family planning counselling for HIV/AIDS clients |  |  |
| [Q7y] Provide condoms for preventing further transmission of HIV |  |  |
| [Q7z] Provide TB testing to HIV clients |  |  |
| [Q7aa] Provide HIV testing to TB clients |  |  |

[Q8] Number of Health Workers:

|  | Number of Full-Time | Number of Part-Time/Voluntary |
| --- | --- | --- |
| [Q8a] Generalist medical doctors |  |  |
| [Q8b] Specialist medical doctors |  |  |
| [Q8c] Nurses |  |  |
| [Q8d] Midwives |  |  |
| [Q8e] Medical Technologists |  |  |
| [Q8f] Barangay Health Workers |  |  |

[Q9] Now I would like to ask you a few questions about waste management practices for sharps waste, such as needles or blades. **How does this facility finally dispose of sharps waste (e.g., filled sharps boxes)?**

[ ] Burn Incinerator: 2 chamber industrial

[ ] Burn Incinerator: 1 chamber drum/brick

[ ] Open Burning: Flat ground – no protection

[ ] Open Burning: Pit or protected ground

[ ] Dump without burning: Flat ground – no protection

[ ] Dump without burning: Covered pit or pit latrine

[ ] Dump without burning: Open-pit no protection

[ ] Dump without burning: Protected ground or pit

[ ] Remove offsite: Stored in covered container

[ ] Remove offsite: Stored in other protected environment

[ ] Remove offsite: Stored unprotected

[ ] Other: ______________________________________

[ ] Never has sharp waste

[Q10] Now I would like to ask you a few questions about waste management practices for medical waste other than sharps, such as used bandages. **How does this facility finally dispose of medical waste other than sharps boxes?**

[ ] Burn Incinerator: 2 chamber industrial

[ ] Burn Incinerator: 1 chamber drum/brick

[ ] Open Burning: Flat ground – no protection

[ ] Open Burning: Pit or protected ground

[ ] Dump without burning: Flat ground – no protection

[ ] Dump without burning: Covered pit or pit latrine

[ ] Dump without burning: Open-pit no protection

[ ] Dump without burning: Protected ground or pit

[ ] Remove offsite: Stored in covered container

[ ] Remove offsite: Stored in other protected environment

[ ] Remove offsite: Stored unprotected

[ ] Other: ______________________________________

[ ] Never has sharp waste

[Q11] Does this facility have any guidelines on standard precautions for infection prevention?

[ ] Yes [ ] No

[Q12] Which of the following are available in the general outpatient area facility today?

|  | Available | Not Available |
| --- | --- | --- |
| [Q12a] Clean running water (piped, bucket with tap, or pour pitcher) |  |  |
| [Q12b] Hand-washing soap, liquid soap |  |  |
| [Q12c] Alcohol based hand rub |  |  |
| [Q12d] Disposable latex gloves |  |  |
| [Q12e] Waste receptacle (pedal bin) with lid and plastic bin liner |  |  |
| [Q12f] Sharps container (“safety box”) |  |  |
| [Q12g] Environmental disinfectant (e.g. chlorine, alcohol) |  |  |
| [Q12h] Disposable syringes with disposable needles |  |  |
| [Q12I] Auto-disable syringes |  |  |

**[Q13] Are the following documents available today?**

|  | Yes | No | Document not yet available |
| --- | --- | --- | --- |
| [Q13a] National HIV Counseling and Testing guidelines |  |  |  |
| [Q13b] National guidelines for clinical management of HIV/AIDS |  |  |  |
| [Q13c] National TB/HIV Collaboration guidelines |  |  |  |
| [Q13d] National ART guidelines |  |  |  |
| [Q13e] National guidelines for PMTCT |  |  |  |
| [Q13f] System/guidelines/policies for diagnosis of HIV among TB patients |  |  |  |
| [Q13g] System/guidelines/policies referral of TB patients to HIV testing |  |  |  |
| [Q13h] System/guidelines/policies referral of HIV patients to TB testing |  |  |  |

**[Q14] Which of the following equipments are available and functional today?**

|  | Available and Functional | Available not Functional | Available, don’t know if functioning | Not Available |
| --- | --- | --- | --- | --- |
| [Q14a] Glass Slide and cover slips |  |  |  |  |
| [Q14b] ELISA washer |  |  |  |  |
| [Q14c] ELISA reader |  |  |  |  |
| [Q14d] Specific assay kit- HIV antibody testing by ELISA |  |  |  |  |
| [Q14e] CD4 counter |  |  |  |  |
| [Q14f] Specific assay kit- CD4 test |  |  |  |  |

**[Q15] Which of the following medicines and commodities are available in this health facility today? (check appropriate column)**

|  | **Observed Available** | | **Not Observed** | | |
| --- | --- | --- | --- | --- | --- |
|  | At least one not expired | Available but expired | Reported available but not seen | Not Available Today | Never Available |
| [Q15a] HIV rapid test kits |  |  |  |  |  |
| [Q15b] Male Condoms |  |  |  |  |  |
| [Q15c] Female condoms |  |  |  |  |  |
| [Q15d] Syringes |  |  |  |  |  |
| [Q15e] Vials |  |  |  |  |  |

**[Q16] Has there been a stock-out of HIV rapid test kits in the past 4 weeks?**

[ ] Yes

[Q16a] IF yes: how many days was the stock out? _________________________

[Q16b] Reason for delay: ________________________________________________

[ ] No

[ ] Facility does not stock HIV rapid test kits

**[Q17] Who is the principal person responsible for managing the ordering of medical supplies at this facility?**

[ ] Nurse [ ] Pharmacy Technician

[ ] Midwife [ ] Pharmacist

[ ] Physician [ ] Others

**[Q18] Which of the following mechanisms is used to determine this facility’s resupply quantities?**

[ ] Facility itself (pull distribution system) [ ] Higher level facility (push distribution)

**[Q19] How are the facility’s resupply quantities determined?**

[ ] Formula (any calculation) [ ] Don’t know

[ ] Other means: ________________________________________________________

**[Q20] Where are the main sources of your medicines and supplies?**

[ ] DOH Central Warehouse [ ] NGOs [ ] LGU warehouses

[ ] Provincial Warehouses [ ] Donors [ ] Others: ________________

[ ] Regional Warehouses [ ] Private Sources ___________________

**[Q21] Who is responsible for transporting products from central medical stores to your facility?**

[ ] Government couriers [ ] This facility collects

[ ] Private/third-party couriers [ ] Others: ________________________________

**[Q22] For the last order, how long did it take between ordering and receiving products?**

[ ] Less than 2 weeks [ ] Between 1 and 2 months

[ ] 2 weeks to 1 month [ ] More than 2 months

**[Q23] Have there been stockouts in TB supplies and commodities in the past three months?**

[ ] Facility does not stock [ ] No

[ ] Yes

[Q23a] How many days was the stock out? _________________________

[Q23b] Reason for delay: ________________________________________________

**For Barangay Health Workers*:**

| **Code Name** | **Highest Educational Attainment (name of degree, year obtained)** | **Trainings, Orientations, Seminars or Worshops attended in the last three years related to TB/HIV (Month, Year, Name of Training, Organizer of Training)** | | | **# of Years as BHW** |
| --- | --- | --- | --- | --- | --- |
|  |  | **Year** | **Title of Training** | **Organizer** |  |

****Please list on a separate sheet of paper***

[Q24] Who has received **training** **or certification** in HIV voluntary counseling and testing in the **last two years**?

[ ] Physician (how many? _______) [Q24a]

[ ] Nurses (how many? _______) [Q24b]

[ ] Midwives (how many? ______) [Q24b]

[ ] Medical technologists (how many? ______) [Q24c]

[Q25] Who has received **refresher courses** in HIV voluntary counseling and testing in the **last one year**?

[ ] Physician (how many? _______) [Q25a]

[ ] Nurses (how many? _______) [Q25b]

[ ] Midwives (how many? ______) [Q25c]

[ ] Medical technologists (how many? ______) [Q25d]

[Q26] Who has received **training** **or certification** in Management of HIV and TB co-infection in the **last two years**?

[ ] Physician (how many? _______) [Q26a]

[ ] Nurses (how many? _______) [Q26b]

[ ] Midwives (how many? ______) [Q26c]

[ ] Medical technologists (how many? ______) [Q26d]

[Q27] Who has received **refresher courses** in Management of HIV and TB co-infection in the **last one year**?

[ ] Physician (how many? _______) [Q27a]

[ ] Nurses (how many? _______) [Q27b]

[ ] Midwives (how many? ______) [Q27c]

[ ] Medical technologists (how many? ______) [Q27d]

**[Q28] Does this facility have an HIV testing accredited/certified medical technologist?**

[ ] No

[ ] Yes

[Q28a] how many full-time/part-time? __________________________

[Q28b] When certification received, from whom? _________________________________

[Q28c] Any refresher courses? ________________________________________

**[Q29] Does this facility have a trained/certified HIV counselor?**

[ ] No

[ ] Yes

[Q29a] how many full-time/part-time? __________________________

[Q29b] When certification received, from whom? _________________________________

[Q29c] Any refresher courses? ________________________________________

**TB/HIV Patient Burden and Referral System**

[Q30] Number of TB patients from March 2015 to March 2016: _____________

[Q31] How many TB patients consented to HIV testing: ________________

[Q32] Of consenting patients, how many were actually tested for HIV in the facility: ____________

[Q33] Of consenting patients, how many were referred for HIV testing to other facilties: _____________

[Q34] Of the total number tested, how many have HIV results in TB register: ______________

[Q35] Of the total number of TB patients, how many have HIV results in TB register: ___________

[Q36] Of those who were not lost to follow-up, how many positive were enrolled in HIV care: __________

[Q37] How many commenced ART during TB treatment: __________

[Q38] Referral mechanism for HIV testing among TB patients:

Nearest HIV testing centers where TB patients are referred: _____________________________

*END OF QUESTIONNAIRE*

**QUANTITATIVE INSTRUMENT 3: SURVEY FOR TB PATIENTS**

Greetings! We would like to ask 15 to 30 minutes of your time to answer the following questions. There are no right or wrong answers and we would appreciate your honesty in answering this questionnaire. Hence, it would really help us if you answer all the questions however, please feel free to leave any questions you do not want to answer. If you have any questions, please do not hesitate to contact the person who gave this questionnaire to you. Thank you!

**Part I. Demographic Information**

[Q1.1] Age: ________

[Q1.2] Sex: [ ] Male [ ] Female

[Q1.3] Civil Status: [ ] Single [ ] Married [ ] Live-in [ ] Widow/er [ ] Separated

[Q1.4] Occupation:

[ ] Government employee

[ ] Private company employee

[ ] OFW

[ ] Business

[ ] Housewife/Homemaker

[ ] Retired

[ ] Others (pls specify: ________________________________________________)

[ ] No occupation

[Q1.5] Highest Educational Attainment:

[ ] Elementary Graduate

[ ] High School Graduate

[ ] College Graduate

[ ] Postgraduate (pls specify: ___________________________)

[ ] Vocational/Technical (pls specify: ___________________________)

[ ] Others (pls. specify _______________________)

[ ] No grade completed

[Q1.6] Approximately how much do you earn per month?

[ ] < P5, 000 per month

[ ] P5,001 to P8,000 per month

[ ] P8,001 to 15,000 per month

[ ] P15,001 to 20,000 per month

[ ] >P 20,000 per month

[Q1.7] What is the distance of your house from nearest health center?

[ ] <1 km

[ ] 1 to 5 km

[ ] 6 to 10 km

[ ] 11 to 20 km

[ ] > 21 km

[Q1.8] How many **minutes** does it take for you to get from your house to your health center? ______

[Q1.9] What do you use as a mode of transport from your house to the health center? *(Check all that apply)* [ ] Walk [ ] Bus [ ] Tricycle [ ] Jeep [ ] Pedicab [ ] Others: _____

[Q1.10] On average, how much is your ***one-way* fare** from your house to the health center? PhP ____

[Q1.11] Start of TB treatment (mm/dd/yyyy): _____ / _____ / ______

[Q1.12] Type of TB: [ ] TB-smear negative [ ] TB-smear positive [ ] Extrapulmonary

[Q1.13] Type of TB patient:

[ ] First-time

[ ] Defaulter (treatment was interrupted for 2 consecutive months or more)

[ ] Relapse/Reinfection

[Q1.14] Have you heard about HIV testing before? [ ] Yes [ ] No

[Q1.15] Source of information about HIV testing:

[ ] Health worker

[ ] Mass Media (TV, radio, newspapers)

[ ] Social Media (Facebook, Twitter)

[ ] Friends

[ ] Family Members

[ ] Work Colleagues

[Q1.16] When you were first diagnosed with TB, were you offered HIV testing?

[ ] No (proceed to Q1.17)

[ ] Yes (answer all succeeding questions

[Q1.16a] Were you given information where to have HIV testing? [ ] Yes [ ] No
 [Q1.16b] Did you consent to HIV testing when you were first offered? [ ] Yes [ ] No

[Q1.17] At what point during TB treatment were you offered HIV testing?

[ ] 1 to 29 days after start of TB drug regimen

[ ] After two months of TB drug regimen

[ ] After three months of TB drug regimen

[ ] After four months of TB drug regimen

[ ] After five months of TB drug regimen

[ ] Towards the end of TB drug regimen

[ ] Never offered HIV testing

[Q1.18] Have you had yourself tested for HIV? [ ] Yes (answer Q1.19) [ ] No (answer Q1.20)

**[Q1.19] For those who answered YES in Q1.18:**

[Q1.19a] Where did you take the HIV test? _______________________

[Q1.19b] How long was the delay between the offer to have yourself HIV tested, and the actual HIV test (in hours, days or weeks)? ________________

[Q1.19c] Reason for the delay (if any, check all that apply):

[ ] Lack of money/time

[ ] Emotional unpreparedness

[ ] Thought HIV testing was not important

[ ] Did not perceive myself at risk of HIV

[ ] Lack of trust in credibility of testing center

[ ] Cumbersome process

[ ] Testing center was far from home

[ ] Fear of stigmatization

[ ] Other reasons: _________________________________________________

***SKIP Q1.20 AND CONTINUE ANSWERING Q.21***

**[Q1.20] For those who answered YES in Q1.18:**

[Q1.20a] Why did you not have yourself tested for HIV?

[ ] Lack of money/time

[ ] Emotional unpreparedness

[ ] Thought HIV testing was not important

[ ] Did not perceive myself at risk of HIV

[ ] Lack of trust in credibility of testing center

[ ] Cumbersome process

[ ] Testing center was far from home

[ ] Fear of stigmatization

[ ] Other reasons: _________________________________________________

[Q1.21] I know somebody who I suspect has died of HIV/AIDS: [ ] Yes [ ] No

Part II. TB/HIV. Below are some statements about TB/HIV. To what extent do you agree with the statements? Put an **X** in the column that represents how you feel.

|  | 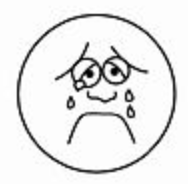  Very Strongly  Disagree | 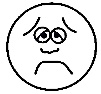  Strongly Disagree | 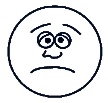  Disagree | 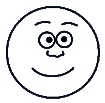  Agree | 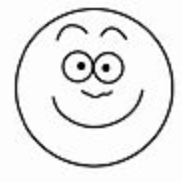  Strongly  Agree | 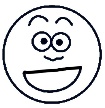  Very Strongly Agree |
| --- | --- | --- | --- | --- | --- | --- |
| 1. In my opinion, there is a strong association between TB and HIV. |  |  |  |  |  |  |
| 1. I think TB infection worsens HIV infection. |  |  |  |  |  |  |
| 1. I think HIV infection worsens TB infection. |  |  |  |  |  |  |
| 1. HIV infection has no symptoms. |  |  |  |  |  |  |
| 1. Provider-initiated HIV counseling and testing among TB patients is important. |  |  |  |  |  |  |
| 1. I believe that the control of tuberculosis helps to control the spread of HIV/AIDS. |  |  |  |  |  |  |
| 1. It is important that TB patients be tested for HIV. |  |  |  |  |  |  |
| 1. A healthy-looking person can have HIV/AIDS. |  |  |  |  |  |  |

Part III. Perceptions of Appropriateness of Delegating HIV testing among TB Patients to
BHWs. Below are series of statements regarding BHWs and their potential contributions to HIV testing and counseling. To what extent do you agree with the statements? Put an **X** in the column that represents how you feel.

|  | 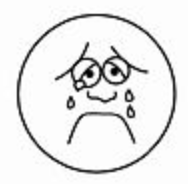  Very Strongly  Disagree | 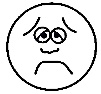  Strongly Disagree | 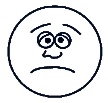  Disagree | 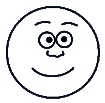  Agree | 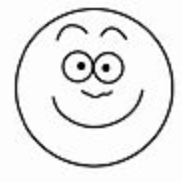  Strongly  Agree | 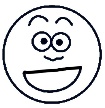  Very Strongly Agree |
| --- | --- | --- | --- | --- | --- | --- |
| 1. I would prefer that **physicians** conduct HIV counseling to me |  |  |  |  |  |  |
| 1. I would prefer that **physicians** conduct HIV testing to me. |  |  |  |  |  |  |
| 1. I would prefer that **nurses** conduct HIV counseling to me. |  |  |  |  |  |  |
| 1. I would prefer that **nurses** conduct HIV testing to me. |  |  |  |  |  |  |
| 1. I would prefer that **medical technologists** conduct HIV counseling to me. |  |  |  |  |  |  |
| 1. I would prefer that **medical technologists** conduct HIV testing to me. |  |  |  |  |  |  |
| 1. I would prefer that **midwives** conduct HIV counseling to me. |  |  |  |  |  |  |
| 1. I would prefer that **midwives** conduct HIV testing to me. |  |  |  |  |  |  |
| 1. I would prefer that **Barangay Health Workers (BHWs)** conduct HIV counseling to me. |  |  |  |  |  |  |
| 1. I would prefer that **Barangay Health Workers (BHWs)** conduct HIV testing to me. |  |  |  |  |  |  |
| 1. I find it easy to build rapport with BHWs hence I would rather have them conduct HIV counseling to me. |  |  |  |  |  |  |
| 1. I find it easy to build rapport with BHWs hence I would rather have them conduct HIV testing to me. |  |  |  |  |  |  |
| 1. I feel that BHWs can better understand my health needs hence it is better to have myself counseled for HIV from them. |  |  |  |  |  |  |
| 1. I would rather have myself tested for HIV from BHWs since they frequently go to the community. |  |  |  |  |  |  |
| 1. I would rather have myself counseled for HIV from BHWs since they frequently go to the community. |  |  |  |  |  |  |
| 1. I would rather have lay people like BHWs conduct HIV counseling to me. |  |  |  |  |  |  |
| 1. I would rather have lay people like BHWs conduct HIV testing to me. |  |  |  |  |  |  |
| 1. I am afraid that BHWs would gossip about my HIV status if they would be the one to do HIV counseling and testing. |  |  |  |  |  |  |
| 1. I would rather test for HIV by myself (self-testing). |  |  |  |  |  |  |
| 1. I would be more likely to have myself tested if HIV screening was done in my own home. |  |  |  |  |  |  |
| 1. I would be more likely to have myself tested for HIV if my entire household would get tested too. |  |  |  |  |  |  |

Would you be willing to participate in a brief interview that will clarify your answers to these questions?

[ ] Yes. Please write your cellphone number so we can contact you in the future (we will keep this private): ____________________________

[ ] No

*END OF QUESTIONNAIRE*

Thank you for your participation!

:)

**QUANTITATIVE INSTRUMENT 4: FEASIBILITY RATING OF PROGRAM MANAGERS**

**Title of Study**: Assessing the Feasibility and Appropriateness of delegating HIV Counseling and Testing among TB Patients to Community Health Workers in select primary health care centers of the Philippines: an implementation research

**Principal Investigator**: Tyrone Reden Sy

**Rationale**: A systematic review in sub-saharan Africa has found that task shifting of HIV care to trained community health workers enhances reach, uptake and quality of HIV services as well as the dignity, quality of life and retention in care of people living with HIV. Their presence was also reported to reduce waiting times, streamline patient flow, and reduce the workload of health workers. More importantly, their presence has also contributed to demystifying HIV in their communities and counteracted social barriers such as stigma which then led to increase in uptake of HIV services.

*Source: Mwai et al, 2013; https://www.ncbi.nlm.nih.gov/pmc/articles/PMC3772323/*

After consultation with various CBOs, CSOs, NGOs, and local program managers, the following financial, infrastructural/operational, and training/technical requirements have been identified in order to make the delegation of HIV counselling to select BHWs feasible in the context of San Jose del Monte City, Bulacan, a Category A area. Please compare the requirements identified below (first column) with the existing contexts in San Jose del Monte City (second column) and score each from **1** (low feasibility) to **4** (high feasibility).

**Your answers to these questions will not be identified as coming from you, and will potentially help you and other program managers approximate the extent of feasibility of this intervention not only in San Jose del Monte but also in other Category A (High TB and HIV prevalence) municipalities with similar geographic and health care workers profile.**

| **Financial Requirements** | **Present Context in San Jose del Monte City, Bulacan (SJDM)** | **Score** |
| --- | --- | --- |
| 1. Additional honorarium or incentives (e.g. hazard pay, travel reimbursement) for BHWs | Honorarium existing; but additional incentives need to be negotiated with LCEs |  |
| 2. Budget for training BHWs (approx. PhP 15,000 to PhP 20,000 trainee) | Training being done but only for doctors, nurses, peer educators; need to be negotiated with LCEs |  |
| **Infrastructure and Operation Requirements** | **Present Context in SJDM** | **Score** |
| 1. Doctors available 5x a week | 17.6% of health facilities (HFs) have full-time doctors (available 5x a week);  82.4% of HFs have part-time doctors (available at least once a week) |  |
| 2. Rooms with visual and/or auditory privacy | 35.3% of HFs have rooms with auditory privacy  41.2% of HFs have room with visual privacy |  |
| 3. Mechanism for infectious waste disposal | Mechanisms for disposal of infectious waste existing in 100% of HFs |  |
| 4. Guidelines/ terms of reference/ responsibilities of select BHWs as provider of HIV counseling | Not yet existing |  |
| 5. Improvement of supply chain and storage for medical supplies (esp. test kits) | 41.2% of HFs reported receiving new supplies 1 to 2 months after placing orders;  29.4% of HFs said it took more than 2 months |  |
| 6. Delineate deaths caused by TB alone and deaths caused by TB/HIV | May be extrapolated from existing TB/HIV databases |  |
| 7. Agency to monitor the implementation of BHWs as HCT providers | TB/HIV collaboration group existing |  |
| 8. Establishing linkages with CBOs and CSOs | SJDM has limited partnerships with CBO/CSO |  |
| **Technical and training requirements** | **Present Context in SJDM** | **Score** |
| 1. Training on how to conduct HIV counseling (e.g. HIV 101, TB/HIV 101, counseling etiquette, HIV laws, HIV reporting forms and linkage to care) | 88.2% of BHWs in health facilities reported being trained on basic HIV knowledge; 23.5% reported being trained on TB/HIV in the last two years; some still have misconceptions about HIV and TB/HIV |  |
| 2. Training on how to use the rapid test kits | Only 5.9% of BHWs in HFs reported being trained on HCT |  |
| 3. Community HIV awareness campaigns | SJDM stakeholders reported conduct of community awareness campaigns |  |
| 4. HIV 101 refresher course for other HRH | Most physicians and nurses trained on HCT in the last two years; less than 10% of midwives in HFs were trained in HCT |  |
| 5. Knowledge/training transfer mechanisms from upper to lower levels of the health system | Capacity building activities available but knowledge transfer mechanisms needs to be more concretely defined |  |
| **BHW Characteristics** | **Present Context in SJDM** | **Score** |
| 1. Time spent for BHW responsibilities less than working time per week | Working minutes per week: 1,920 mins  Time spent for responsibilities: 1,575 mins |  |
| 2. BHWs are high school graduates | 71% of BHWs are high school graduates |  |
| 3. Few stigmatization behaviors towards TB/HIV patients among BHWs | Only 1 in 5 BHWs have hesitations in talking to a patient with TB/HIV |  |
| 4. Good knowledge in patient counselling principles among BHWs | Only 16.5% of BHWs have adequate knowledge; 69.1% needs refresher course |  |

Thank you for your time!
